# Ab initio investigation of a hypersonic double cone experiment

**DOI:** 10.1126/sciadv.ads2147

**Published:** 2025-02-05

**Authors:** Maninder S. Grover, Paolo Valentini, Nicholas Bisek

**Affiliations:** ^1^ Air Force Research Laboratory, Wright-Patterson Air Force Base, Dayton, OH 45433, USA.; ^2^ Air Force Research Laboratory, Kirtland Air Force Base, Albuquerque, NM 87123, USA.

## Abstract

This article presents a direct molecular simulation (DMS) of a reactive Mach 8.2 oxygen flow over a double cone geometry. The free stream conditions and article configuration generate a flow with thermal and chemical nonequilibrium, which are common attributes of hypersonic flight. This scenario was first studied experimentally at Calspan University of Buffalo Research Center’s test facility. DMS is a particle method that uses quantum mechanically derived interaction potentials to simulate molecular collisions within a flow field. Since these interaction potentials are the only modeling inputs used in the simulation, all flow features can solely be attributed to the ab initio potential energy surfaces. Hence, providing a comparison of a hypersonic ground test and numerical data anchored to quantum mechanics. The fundamental nature of DMS is leveraged to investigate molecular level mechanisms prevalent in the flow, and comparisons with lower fidelity simulations are presented to highlight the role of these first principles calculations as benchmark solutions.

## Teaser

Quantum mechanically guided simulation models a hypersonic ground test.

## INTRODUCTION

Hypersonic flight leads to the formation of strong shock waves where the post shock temperatures can easily exceed a few thousand Kelvin. Such an energetic environment leads to the excitation of internal energy modes and causes chemical reactions in the shock-heated gas. Because of the high velocity and low density associated with these flows, the rate of thermodynamic and reactive processes competes with the characteristic flow time potentially causing the flow to be in thermochemical nonequilibrium (*1*). In addition, complex vehicle geometries can lead to multiple shocks to form along the vehicle. These shocks can intersect with each other (shock-shock interactions) and impinge on the flow boundary layer causing mechanical nonequilibrium in the flow field (*2*). Because of the coupling between thermodynamics, chemistry, transport phenomena, and fluid mechanics, accurate predictive simulation of these flows is challenging.

The most prevalent methodology to simulate nonequilibrium hypersonic flows is by using “multitemperature” models within the framework of Navier-Stokes computational fluid dynamics (CFD). In this approach, the average energy in each internal energy mode is represented as a separate “temperature.” Relaxation laws are used to couple internal energy modes with the translational mode. Chemical reactions in these simulations are modulated to account for thermal nonequilibrium using a “rate controlling temperature,” which is typically an empirical formulation of internal and translational temperatures. Among the multitemperature models, the two-temperature (2T) models (*3*–*7*) are the most common. These models assume equilibrium between the translational and rotational energy modes while separately tracking the evolution of the average vibrational energy of the gas. In general, multitemperature models are computationally efficient but lack accuracy as empirical modeling assumptions used to formulate the rate controlling temperature may not be applicable over a wide range of nonequilibrium scenarios (*8*, *9*). These models often rely of experimentally obtained reaction rate and transport coefficients. At high temperatures seen in hypersonic flight, typically in excess of ∼5000 K, experimental data becomes difficult to obtain and carries considerable uncertainties (*10*–*19*). Therefore, CFD simulations using this paradigm propagate uncertainties arising from underlying experimental database, extrapolation of experimental data, and empirical modeling assumptions. Experimental campaigns (*20*–*22*) using ground testing have been conducted to help validate existing computational models. Once again, because of challenging flow conditions and very short observation times (sometimes only fractions of a millisecond), these experiments tend to be poorly characterized and have notable test and measurement uncertainties (*20*, *23*–*25*). As a result, disagreements between experimental observations and CFD results cannot be exactly attributed to uncertainties in nonequilibrium model formulations, rate coefficients, or even test conditions.

Advances in computational chemistry have enabled empiricism-free, quantum mechanically guided investigations into internal energy excitation, chemical reactions, and transport phenomena of gases. Not bound by limitations of experimental hardware, these theoretical approaches complement experimental data by providing data at conditions that would be difficult to capture experimentally. The first step in this approach is to generate an interaction potential for the gas species of interest. This is done by solving the electronic Schrödinger equation for thousands of geometric arrangements of a molecular system to calculate its potential energy. These single-point energies are then interpolated using sophisticated polynomial functions (*26*–*32*) or neural-network interpolations (*33*) to generate a potential energy surface (PES). Gradients from these PESs are used to simulate molecular collisions or trajectories (*34*).

Billions of these molecular trajectories can be calculated at specific temperatures to generate databases of molecular state transitions. These state transition databases form the foundation of the state-to-state (StS) approach (*35*–*39*), which treats internal energy states of a particle as separate pseudo-species. The population transfer between each internal state is carried out by solving the master equation (*40*) using the ab initio state transitions databases, and the mass density of a specie is determined by the sum of the population in each internal energy state. The StS method provides an empiricism free way to study thermochemistry of a system, and recent research has also extended the StS method to be integrated in into CFD solvers (*41*–*43*). However, the StS approach depends on state transition datasets, which must be calculated a priori, and any gas state not accounted for in the dataset is extrapolated in a StS-CFD calculation. In a nonequilibrium flow field simulation that can have many combinations of internal and translational energy distributions, extensive interpolation outside of the calculated dataset is required. Furthermore, for molecule-molecule collisions (N_2_ + N_2_, O_2_ + O_2_ ... etc.) there can be up to **O**(10^15^) possible state transitions. Computing state transition rates and performing master equation analysis for such a large magnitude of possible transitions are intractable with current computer hardware. As a result, StS approaches rely heavily on simplification strategies, such as assuming equilibration between translational and rotational modes or binning of internal energy states (*38*, *44*–*46*) to resolve molecule-molecule interactions. It has been shown that binning of internal energy states leads a loss of information about relaxation between internal states, which consequently can lead to variation in macroscopic flow variables depending on the simplification strategy used (*47*). Hence, these assumptions and interpolations add a degree of uncertainty to StS-CFD solutions.

Here, we use the direct molecular simulation (DMS) method (*48*). DMS evolved from the CTC-DSMC method of Koura and coworkers (*49*–*51*) and is the first principles variant of the direct simulation Monte Carlo (DSMC) method and embeds the molecular trajectory calculations directly within a time accurate flow simulation. In this manner, DMS by-passes the need to calculate state transition datasets or binning assumptions made by StS calculations. In a DMS calculation, all internal state transitions compatible with the thermodynamic state of the gas are permitted, and all flow features are directly attributed to the molecular interactions being simulated using a quantum mechanically derived PESs. Therefore, DMS provides a unified framework where all flow physics such as transport properties, reaction rates, internal energy excitation rates, etc. can be attributed to a single modeling input—the PES. This makes DMS ideally suited to benchmark lower fidelity models. Because of the high computational cost associated with calculating a large amount of molecular trajectories, in the past, DMS has only been limited to simple problems such as zero-dimensional (0D) reactors to study molecular level excitation and dissociation mechanisms (*52*–*57*), 1D shock waves (*58*), and 2D canonical flows (*19*, *59*–*63*). With growth in computational capabilities, we are now able to scale DMS to domain sizes than can be replicated in ground tests. Here, we present a DMS calculation of a near-continuum, hypersonic, reactive flow that was studied experimentally (*20*), providing a comparison between a hypersonic ground test and numerical data whose origin is solely traceable to quantum mechanics.

**Fig. 1.**
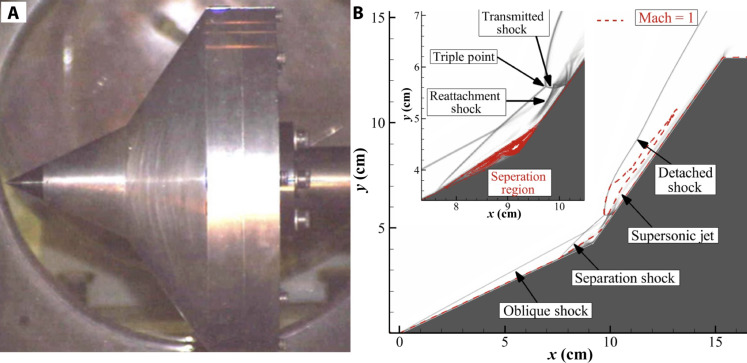
Test article and key flow features of the hypervelocity ground test. (**A**) Test article in the CUBRC test facility before the experiment (*20*). (**B**) Numerical shadowgraph obtained from the DMS calculation, highlighting key flow features and the sonic line (Mach = 1) on the flow field. The inset in (B) provides a magnified view of the biconic junction, labeling important features that form due to shock-shock interactions.

This article focuses on a DMS solution of Run 88 (*23*) conducted at Calspan University of Buffalo Research Center (CUBRC) LENS facility (*20*). This experiment studied a Mach 8.2 oxygen flow over a double cone geometry. Figure 1A shows the test article at the CUBRC test facility (*20*), and Fig. 1B shows the shadow graph obtained from the DMS calculation for this case and highlights key flow features. The inset figure provides a magnified view of the biconic junction. The biconic junction contains a region of subsonic separated flow. When the supersonic gas in the shock layer contained by the oblique shock formed at the tip of the article encounters this region, another oblique shock is formed at the foot of the separation region. This shock impinges on the boundary layer formed on the first cone and is named “separation shock.” Downstream of the separation region, as the boundary layer reattaches another oblique shock named “reattachment shock” is formed. At the same time, the second cone forms detached bow shock, which is intercepted by the separation shock at the “triple point.” The red dashed line in the figure represents the sonic line, and it can be seen that a small region of subsonic flow is formed downstream of the triple point. The shock-shock interaction at the triple point leads to the formation of the transmitted shock, which impinges on the second cone surface. This point of impingement corresponds with the maximum pressure and thermal loading on the test article. Downstream of the transmitted shock, the compressed supersonic gas rapidly expands leading to the formation of a supersonic jet. This jet is bound by the cone and the subsonic region generated by the detached shock.

Figure 1B demonstrates that this flow configuration leads to a complex fluid dynamics system, which includes features such as shock-shock and shock boundary layer interactions. In prior work (*64*), we have studied this configuration using DSMC. This investigation provided insight on how the energetic shock waves cause internal energy excitation and chemical reactions, with sections of flow remaining in thermal and chemical nonequilibrium. It was observed that the chemical kinetics of the flow affects the state of the shocked gas and influences the strength of the shocks and in turn the location and magnitude of peak heating experienced by the article. In this manner, this configuration provides a stringent test case where the fluid mechanics, transport phenomena, and thermochemistry are tightly coupled. For this reason, a DMS of this scenario can serve as a valuable benchmark for lower fidelity models. A DMS of this scale and complexity represents one of the single largest calculations ever performed in the history of fluid mechanics, at an unprecedented level of fidelity. This simulation was run using 1024 nodes containing 64 cores each at Argonne National Laboratory’s Theta machine and required ∼1830 hours of computational time, resulting in ∼120 million core hours of compute resources. While the simulation represents the very cutting edge of what is possible with today’s computational resources, it paves the way for a first principles approach to reactive fluid mechanics.

Here, we first discuss ab initio flow field results obtained from DMS. This is done by summarizing the thermochemical state of the gas in the DMS generated flow field and investigating molecular level mechanisms prevalent in the flow. Then, we provide comparative analysis with a tuned DSMC simulations to assess the DSMC method’s accuracy. Thermochemical and transport cross sections of the DSMC calculation are tuned to prior DMS data for O_2_ + O_2_ and O_2_ + O collisions, for an appropriate comparison with DMS. In previous work, we analyzed CUBRC’s Run 88 only using dissociation rate coefficients that included the electronic excitation factor ϕ_e_ (*64*). In this work, we provide two DSMC solutions, one where the dissociation rates include the electronic excitation factor ϕ_e_ = 16/3 and one without. This allows us to assess the effect of electronic excitation against a benchmark ground state solution. Last, we compare DMS with Navier-Stokes CFD calculations in the literature. Table 1 lists modeling assumptions made by each computational technique, highlighting the fidelity of the computational methods.

**Table 1.** Comparison of modeling inputs for DMS, DSMC, and Navier-Stokes CFD. This table highlights the modeling assumptions and therefore fidelity of the computational techniques discussed in this article.

DMS	DSMC	CFD
1. Ab initio PES	1. Collision cross section model	1. Decoupling between rotational and vibrational energy modes
2. Dilute gas	2. Relaxation cross section model	2. Internal energy relaxation models
	3. Reaction cross section model	3. Reaction rate internal energy coupling model
	4. Dilute gas	4. Coupling between internal energy and reactions
		5. Diffusion coefficients
		6. Thermal conductivity coefficients
		7. Lack of thermal diffusion for multispecies mixtures
		8. Lack of baro-diffusion for multispecies mixtures
		9. Stokes’ hypothesis

### Simulation setup

**Fig. 2.**
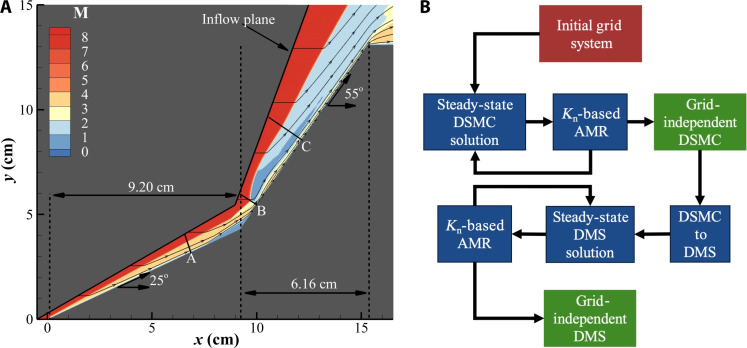
Simulation domain and calculation overview. (**A**) Illustrates the dimensions of the test article and highlights the simulation domain used for DMS and DSMC calculations presented in this article. (**B**) Various steps undertaken to produce the results obtained in this work. The green boxes illustrate the steps where the samples for the final steady state solution are collected.

Figure 2A shows the simulation domain and test article geometry along with Mach contours obtained from the steady-state DMS simulation. Lines marked A, B, and C represent wall normals that are sampled and discussed later in the article. All simulations presented in this article are carried out assuming axis symmetric flow. A cell-dependent particle weight is not practical for axis symmetric simulations as it leads to very few particle counts near the symmetry axis and the number of particles grows radially. This can lead to a configuration where it is possible to have fewer particles than required to determine the thermodynamic state of the gas near the symmetry axis and more than necessary count of particles toward the edge of the domain. To circumvent this behavior, we use a per-cell weighting scheme, where each cell in the domain is assigned a weight proportional to the distance of its midpoint from the axis of symmetry. Particles are then cloned or removed at each time step to maintain the correct local mass density. The article surface is assumed to be isothermal. Because of the short experiment run time (*20*), the wall temperature is assumed to be *T*_w_ = 300 K. Because of the high density and pressure at the article’s surface in this case, it is appropriate to assume full thermal and momentum accommodation of the particles colliding with the double cone surface (*64*–*66*). This is achieved by assuming diffuse reflection (*65*, *66*) of particles striking the surface (α = 1). The article surface is assumed to be chemically inert, and no wall-driven chemistry is modeled.

Nompelis *et. al* (*23*) were the first to conduct CFD analysis of the test facility and the test conditions. They interpreted the experimental free stream to exhibit slight vibrational nonequilibrium, with the free stream temperature as $T_{t,\infty }=T_{r,\infty }=570\;K$ and $T_{v,\infty }=678\;K$. In this work, we assume $T_{t,\infty }=T_{r,\infty }=T_{v,\infty }=606\;K$, which is the average of the translational, rotational, and vibrational temperatures determined by Nompelis *et. al* and weighted by the degrees of freedom of each mode. The free stream conditions for the flows simulated in this work are defined as ρ_∞_ = 1.06 g/m^3^, *u*_∞_ = 3853 m/s, and *T*_∞_ = 606 K. The free stream gas composition was set to the conditions prescribed in (*23*) with the mole fraction of atomic oxygen ζ_O_ = 0.05 and molecular oxygen ζ_O2_ = 0.95.

DSMC simulations were initiated with a uniform grid system with cells 3.4 × 10^−4^ m. Once the solution reached a steady state, stochastic parallel rarefied-gas time-accurate analyzer’s (SPARTA’s) adaptive mesh refinement (AMR) routines are used to adapt the grid such that the grid is refined in regions where the ratio between local mean free path and the cell size in λ/Δ*x* ≤ 1 and coarsen where λ/Δ*x* ≥ 2. Once the new AMR grid is generated, the solution is allowed to reach a steady state, and flow characteristics are analyzed. This process is repeated until the quantity of interest, which in the case of this simulation is the heat flux at the surface, is observed to be independent of the grid. This approach is consistent with recent DSMC analysis of the same scenario demonstrated by our group (*64*). In that study, we presented a coarse grid simulation with ∼2.2 × 10^7^ cells and ∼4 × 10^8^ simulated particles obtained by using seven levels of AMR refinement on the baseline grid system as well as a fine grid simulation with ∼3 × 10^7^ cells and ∼9 × 10^9^ simulated particles obtained using nine levels of AMR refinement on the baseline grid. It was demonstrated that both simulations yielded the identical heat flux experienced by the surface and flow field features such as the locations of shock-shock interactions and the size of the separation region (*64*). The simulations carried out in (*64*) are tuned DSMC simulations where the dissociation rate coefficients include the electronic excitation (ϕ_e_). In addition to the published DSMC solution, a DSMC solution where ϕ_e_ was not included is also presented in this work. The authors would like to clarify that the AMR process was carried out for both DSMC cases presented in this work until a grid-independent solution was obtained. The first half of Fig. 2B sows the procedure used to obtain grid-independent DSMC solutions. The final DSMC simulations were sampled over 20,000 time steps at a frequency of 100 time steps.

The baseline DSMC solution with ϕ_e_ using the coarse grid is taken as a starting point for the DMS solution. Because the DSMC simulation uses collision cross sections tuned to DMS and showed grid invariance at seven levels of AMR, it is reasonable to assume that the DMS simulation is grid invariant at seven levels of AMR, as in a control volume, both these methods will simulate equivalent number of particle “collisions” to determine the thermodynamic state of the gas. A DSMC-to-DMS algorithm is implemented to the modified SPARTA code such that a DMS calculation can be initialized from a DSMC solution. This capability allows us to conserve computational resources associated with the transient flow. The DMS solution is allowed to reach a steady state based on the collision dynamics predicted by the PES. Then, the grid adaption process using AMR described above is repeated while keeping the maximum level of AMR refinements fixed at seven until a new steady state is achieved. The steady-state DMS solution is sampled over 2000 time steps every 200 time steps to generate results shown in this work. The DMS simulation contains ∼2.9 × 10^7^ cells and ∼4.1 × 10^8^ simulated particles, with ∼3.1 × 10^7^ molecular trajectories being calculated every time step. Figure 2B shows the outline of the procedure used to obtain the DMS solution. It was observed that the DSMC calculations were ∼1000× less computationally intensive than the DMS.

## RESULTS

### DMS flow field

#### Thermal and chemical state

**Fig. 3.**
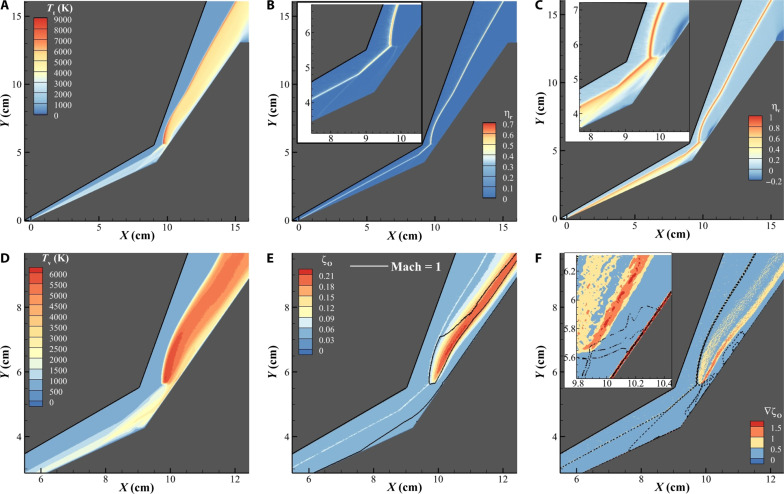
Thermochemical state of the flow field. (**A**) Contour plot for the translational temperature across the simulation domain. (**B**) Contour plot for the degree of rotational nonequilibrium. (**C**) Contour plot for the degree of vibrational nonequilibrium. (**D**) Vibrational temperature contours in the vicinity of the biconic junction. (**E**) Contour plot showing mole fraction of atomic oxygen in the vicinity of the biconic junction. (**F**) Contour plot showing the gradient magnitude of atomic oxygen mole fraction. The inset on (F) shows a magnified view of the contour near the surface of the second conic section.

Figure 3 shows the thermodynamic state of the gas in the flow field. Figure 3A shows the translational temperature of the gas, the peak translational temperature is observed to be at the intersection of the transmitted and reattachment shock ∼*T*_t_ = 9000 K. Here, we describe the degree of nonequilibrium of an internal mode *i* as

$$\eta_{i}=\frac{T_{t}-T_{i}}{T_{t}}$$ (1)

where *T*_t_ is the translational temperature and *T*_*i*_ is the temperature of the internal mode “*i*.” Figure 3B shows the rotational nonequilibrium in the flow field, with the inset providing a magnified view of the biconic junction. The translational and rotational modes are in equilibrium in the flow field, except at shock interfaces. The rotational nonequilibrium is stronger at the free stream-shock interface due to the larger temperature gradients than at shock interfaces contained within the shock layer.

Figure 3C shows the degree of vibrational nonequilibrium. As with the rotational mode, vibrational nonequilibrium is observed at all shock interfaces. The flow over the first conic section is observed to be in vibrational nonequilibrium with η_v_ > 0 for the large section of the flow. This can be explained with the relatively lower post-shock temperatures ∼*T*_t_ = 2200 K as seen in Fig. 3C and fast supersonic flow as seen Fig. 2A in this region. This figure demonstrates that the characteristic flow time for the gas traveling over the first conic section is faster than the vibrational relaxation rate in this region, causing the flow to be in thermal nonequilibrium. Flow over the second conic section is largely in vibrational equilibrium. In this region, higher flow temperatures, lower flow velocity, and the presence of atomic oxygen (*57*) aid in faster relaxation of the vibrational mode. Additionally, downstream of the transmitted shock, in the supersonic jet, we observe η_v_ > 0. In this region, rapid expansion of the flow cools the translational mode, while the vibrational temperature remains high, leading to a region where *T*_v_ > *T*_t_. Note that by definition, η_v_ is always less than one, however, as the vibrational mode is slow to relax and relatively high temperatures observed at the triple point and the detached shock this value approaches one. Figure 3D shows the vibrational temperature field near the biconic junction, with a peak vibrational temperature of ∼*T*_v_ = 6000 K downstream of the triple point in the detached shock layer.

Figure 3E shows the mole fraction of atomic oxygen in the flow field. The strong detached shock and shock-shock interactions downstream of the triple point cause molecular oxygen in the free stream to dissociate. The black solid line in Fig. 3E shows the sonic line, this line indicates that the production of atomic oxygen due to dissociation occurs in the subsonic region downstream of the triple point, and the supersonic jet that forms downstream of the transmitted shock generates a flow feature that shields the test article surface from atomic oxygen by the energetic shocks. In addition, Fig. 3E shows some increase in atomic oxygen along the free stream-shock interfaces. As noted above the free stream of the scenario has 0.05 mole fraction of atomic oxygen. At the free stream-shock interface, the marked increase in pressure and temperature causes pressure and temperature gradient-induced diffusion of atomic species, causing a local increase in atomic oxygen concentration at the interface. These effects are usually ignored in conventional Navier-Stokes CFD; however, because of the fundamental nature of DMS calculations, all transport phenomena are inherently simulated.

Figure 3F shows the spatial gradient magnitude of atomic oxygen mole fraction. In a reactive flow field, this quantity provides an indication of regions where a chemical species may be forming. Dashed lines in Fig. 3F show pressure contours in the flow field; these data are added to the plot to show the various shock structures that form in the flow field along the gradient magnitude of atomic oxygen mole fraction. From this plot, two key locations of atomic oxygen formation become clear: first, at the triple point where the detached shock and the oblique shock intersect. The second and the more prominent streak of the atomic oxygen mole fraction gradient emanates from the location where the transmitted and reattachment shock intersect, hence showing that the strength of the transmitted and the reattachment shock drive the chemistry in the flow. The inset in Fig. 3F provides a magnified view of the contour near the surface of the second conic section. This inset highlights a sharp gradient of atomic oxygen mole fraction near the double cone surface. In this region, sharp temperature gradients seen in the boundary layer induce diffusion (Soret effect) (*67*) of atomic species away from the article surface.

#### Molecular level details

**Fig. 4.**
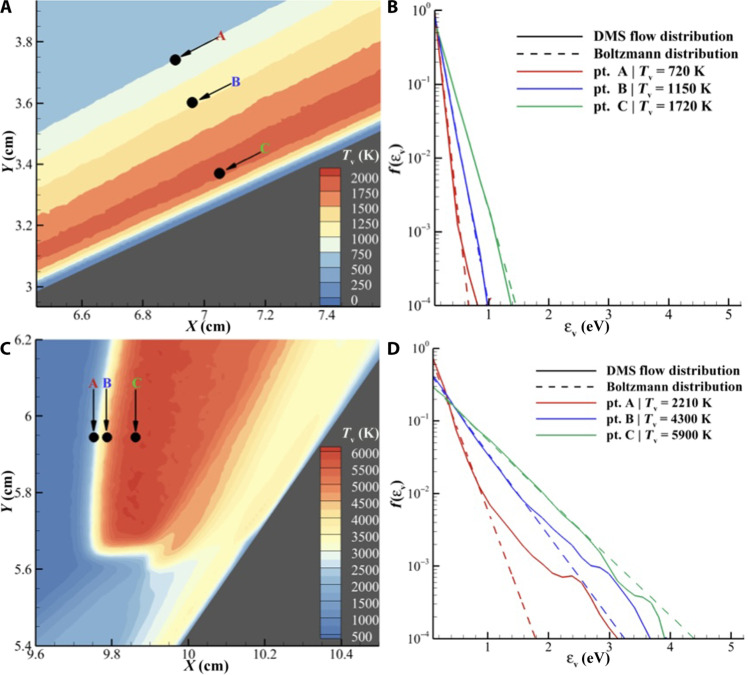
Vibrational distributions at selected points. (**A**) Probe locations along the oblique shock where particle data were collected to construct energy distribution functions. (**B**) Vibrational distribution at points shown in (A). (**C**) Probe locations along the detached shock where particle data were collected to construct energy distribution functions. (**D**) Vibrational distribution at points shown in (C).

As DMS is a particle method, particle information at selected points can be collected to understand the underlying molecular level behavior of the gas. As only the vibrational mode of the gas is observed to be in notable nonequilibrium, molecular population distributions of vibrational energy are studied. Figure 4A shows the vibrational temperature contours for a portion of the flow along the first conic section. Three locations are marked across the oblique shock where particle data are accumulated, and Fig. 4B shows the vibrational energy distribution at these points. It can be seen that as the shock-heated gas vibrationally excites across the weak oblique shock, the vibrational distribution agrees with the expected Boltzmann distribution of the corresponding temperature. Therefore, although in this region vibrational nonequilibrium is observed in Fig. 3C (*T*_t_ ≠ *T*_v_), a two temperature Boltzmann description of the gas is adequate to describe the behavior of the shock layer.

Figure 4C shows the vibrational temperature contours in the vicinity of the triple point. Three locations are marked across the detached shock where particle data are accumulated, and Fig. 4D shows the vibrational energy distribution along at the selected points. It can be seen that during vibrational excitation at points A and B, the vibrational energy distribution has a non-Boltzmann character where the high energy tail is overpopulated when compared to the Boltzmann distribution corresponding to vibrational temperature at these points. This is similar to excitation behavior observed in 0D DMS studies of the oxygen system (*57*). At these points, not only is the gas in thermal nonequilibrium as shown in Fig. 3C, at the molecular level, the gas deviates from Boltzmann (equilibrium) description, compounding the nonlinearity in expected gas behavior in this region. As molecules occupying vibrationally excited states are more likely to dissociate, such a population distribution would lend to a higher dissociation rate than anticipated by a Boltzmann gas.

At point C, where oxygen dissociation occurs (see Fig. 3E), the vibrational energy distribution is observed to be depleted at higher vibrational levels when compared a Boltzmann distribution of the corresponding vibrational temperature. This is because the rate of inelastic kinetic processes responsible to populate the high vibrational energy levels get out competed by the state-specific dissociation rate for vibrationally excited molecules. This phenomenon coupled with the high local flow speed results in this station having a depleted non-Boltzmann distribution. Such a non-Boltzmann distribution will lead to a lower dissociation rate than is expected for a corresponding Boltzmann gas. Point C exemplifies the fact that even when macroscopically a large degree of nonequilibrium is not observed (η_v_ = 0.07 per Fig. 3C), molecular level nonequilibrium could still persist in high speed reactive flows. Non-Boltzmann behaviors observed in Fig. 4D have been previously seen in 0D DMS studies of the oxygen system (*57*). This lends weight to simplified low fidelity models developed on the basis on coefficients and molecular level behaviors observed in 0D reactor simulations (*7*). Similar behaviors have also been observed in studies of nitrogen excitation and dissociation in hypersonic flows using DMS (*60*–*63*) and StS methods (*41*–*43*, *45*).

To generate molecular distributions, attributes of particles are collected in the simulation cell at the selected region. The particle data were accumulated over 6600 time steps in intervals of five time steps to allow randomization of collected samples through advection and molecular collisions. The discretization of the vibrational energy bins for these distributions is consistent with the vibrational energy levels obtained from the diatomic oxygen PES included in the ab initio PESs (*30*–*32*). For reference, this vibrational level database is listed in the table S3.

### Comparison with low fidelity DSMC

In this section, we compare the DMS solution to two DSMC simulations. Details of the DSMC simulations are listed in Methods. Briefly, the two DSMC simulations use identical cross section parameters tuned to DMS data, with the distinction that one simulation does not include the adjustment of the dissociation rate coefficients to account for electronically excited states, labeled as “Tuned DSMC w/o ϕ_e_.” The second simulation assumes full electronic excitation when considering the dissociation rate coefficients, labeled as “Tuned DSMC w/ ϕ_e_.” This paradigm points out the limitation of first principles data in the literature, as only PESs to describe the interactions of O_2_ + O_2_ and O_2_ + O collisions in the electronic ground state are available. To this end, the tuned DSMC simulation w/o ϕ_e_ represents an apples-to-apples comparison with DMS, as the DMS calculation only uses the ground-state PESs, whereas the tuned DSMC w/ ϕ_e_ shows the variation in simulation results given the current state of computational chemistry data. As the flow in the vicinity of the biconic junction contains the most complex features, the flow field results will focus on comparisons in this region.

**Fig. 5.**
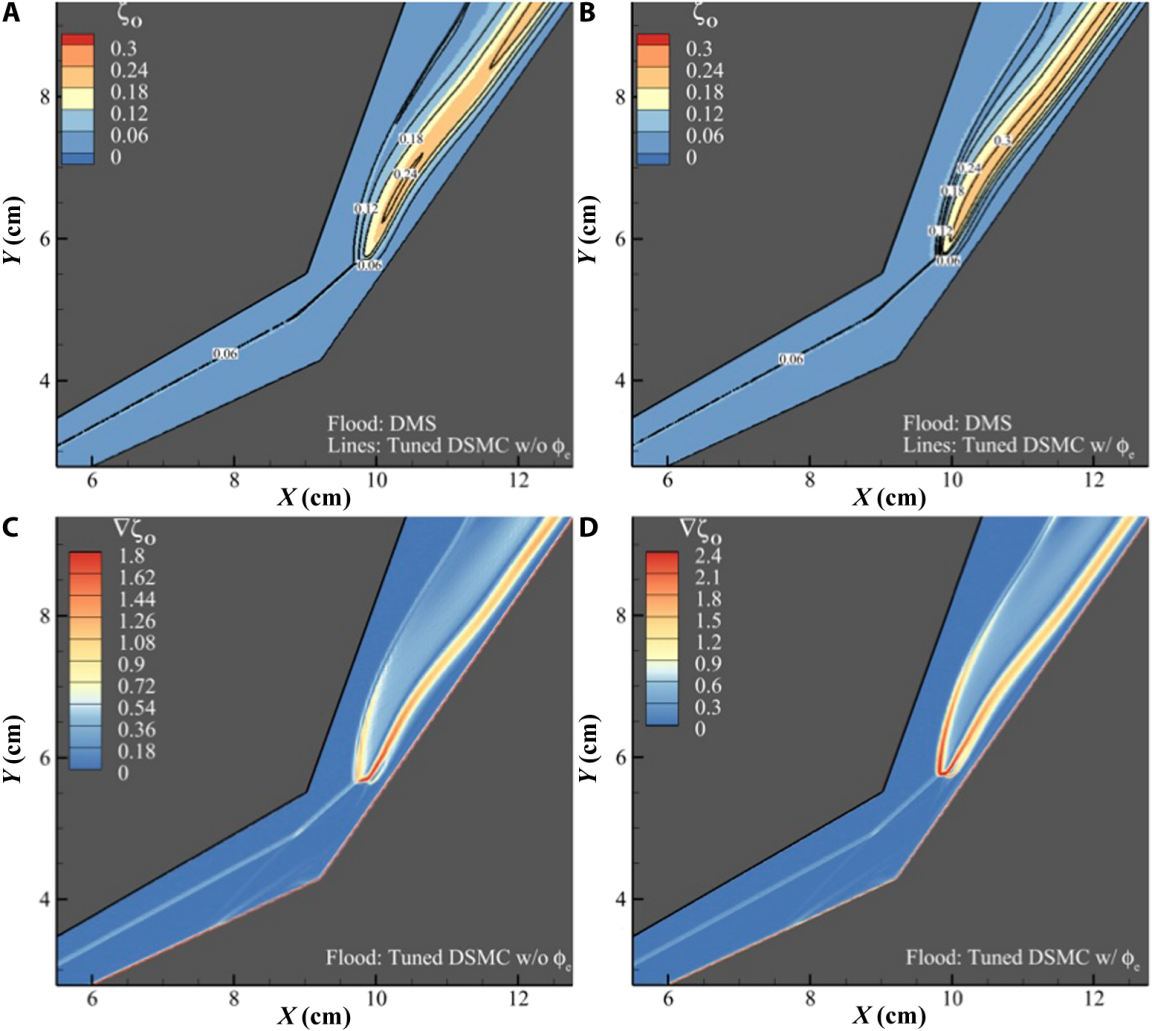
Comparison of chemical activity in the flow field between DMS and DSMC. (**A**) Contour of atomic oxygen mole fraction obtained from DMS (color flood) overlayed with the contour of atomic oxygen mole fraction obtained from DSMC without ϕ_e_ (lines). (**B**) Contour of atomic oxygen mole fraction obtained from DMS (color flood) overlayed with the contour of atomic oxygen mole fraction obtained from DSMC with ϕ_e_ (lines). (**C**) Contour of the atomic mole fraction gradient for the DSMC calculation that excludes the electronic excitation factor ϕ_e_. (**D**) Contour of the atomic mole fraction gradient for the DSMC calculation that includes the electronic excitation factor.

Figure 5A shows a good qualitative agreement between DMS and DSMC without ϕ_e_. As the simulation with the electronic excitation factor increases the dissociation rate by a factor of ϕ_e_ = 16/3 (*57*), we observe a higher mole fraction of atomic oxygen in the DSMC with ϕ_e_ when compared to DMS in Fig. 5B. In addition, all three simulations show a slight increase in atomic oxygen mole fraction at the free stream-shock interface due to diffusion induced by steep temperature and pressure gradients.

Figure 5 (C and D) shows the mole fraction gradient of atomic oxygen in the DSMC calculations. As the total collision energy (TCE) model lacks the appropriate dissociation bias for vibrationally excited molecules, these DSMC simulations produce atomic oxygen at the bow shock where the translational temperature peaks in addition to the region of shock-shock interaction between the transmitted and reattachment shocks, whereas the DMS calculation (Fig. 3F) largely produces atomic oxygen downstream of the transmitted and reattachment shock intersection. This figure highlights how modeling assumptions in lower fidelity simulations can attribute chemical activity to flow features that may not be necessarily generate conditions required for the chemical reactions.

**Fig. 6.**
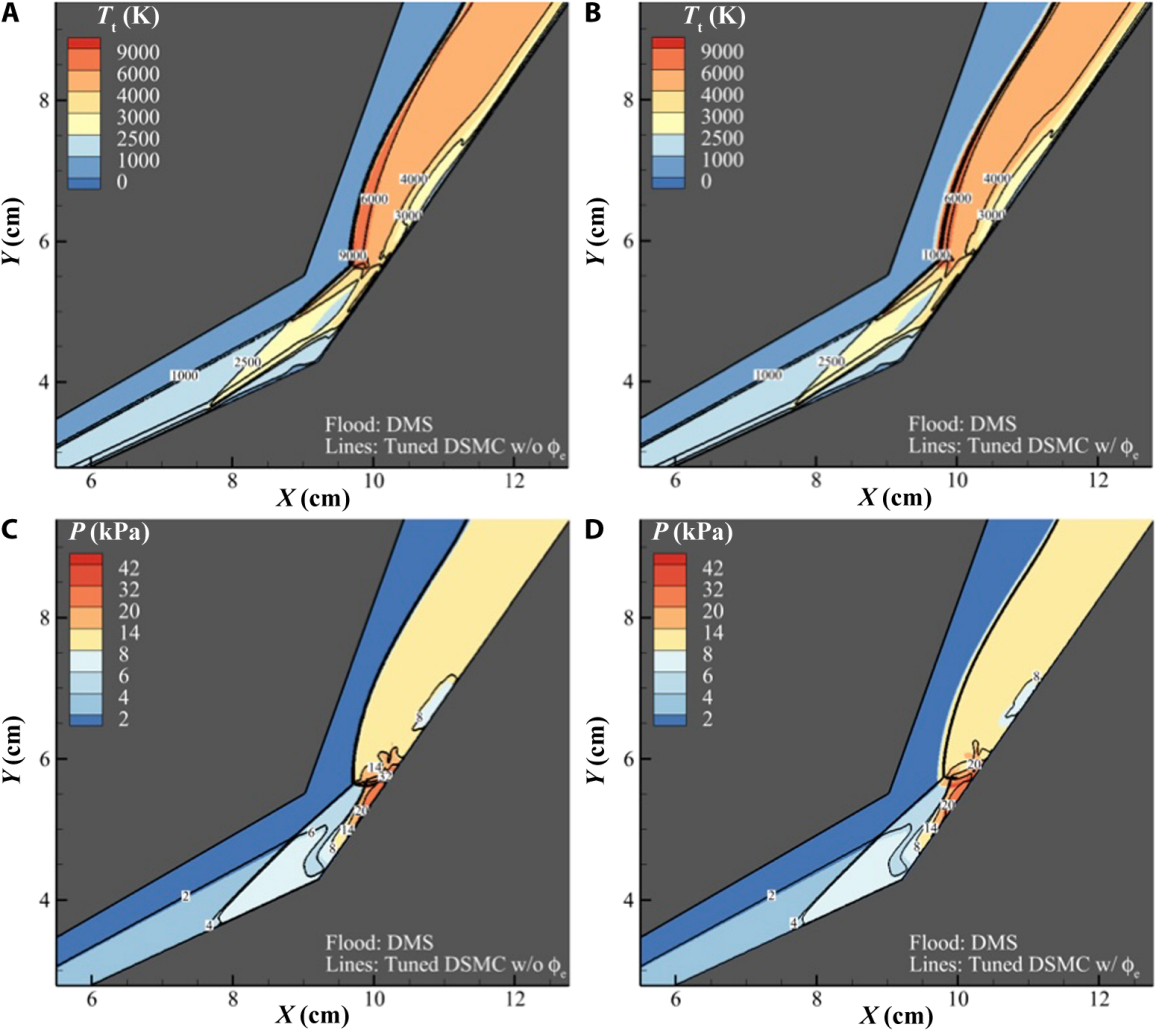
Comparison of temperature and pressure in the flow field between DMS and DSMC. (**A**) Contour of translational temperature obtained from DMS (color flood) overlayed with the contour obtained from DSMC without ϕ_e_ (lines). (**B**) Contour of translational temperature obtained from DMS (color flood) overlayed with the contour obtained from DSMC with ϕ_e_ (lines). (**C**) Contour of the pressure field obtained from DMS (color flood) overlayed with the pressure field contour obtained from DSMC without ϕ_e_ (lines). (**D**) Contour of the pressure field obtained from DMS (color flood) overlayed with the contour obtained from DSMC with ϕ_e_ (lines).

Figure 6A compares translational temperature and pressure contours obtained from DSMC and DMS. Figure 6A shows that translational temperatures obtained from DSMC simulation not using ϕ_e_ provide good qualitative agreement with DMS. Figure 6B shows that the temperature contours of the DSMC simulation with ϕ_e_ agree with DMS on the first conic section, where chemical activity is negligible. However, the DSMC simulation with ϕ_e_ predicts lower temperatures downstream of the triple point. This is consistent with the higher dissociation seen in Fig. 5B as the chemical process of dissociation requires energy to break chemical bonds leading to a lower post-shock temperature.

Figure 6 (C and D) shows the comparison of pressure contours between DMS and DSMC. The pressure contours are useful in highlighting location of key flow features. Along the first conic section the DMS and DSMC pressure contours agree well, all three simulations predict the same location for the separation foot and the separation shock. However, the flow features between the two DSMC simulations diverge downstream of the triple point. Figure 6C shows excellent agreement between the tuned DSMC and DMS for the location of the triple point, transmitted shock, and the point at which the transmitted shock impinges on the test article. Figure 6D shows that for the simulation with ϕ_e_ all the above listed features move further downstream. This is because the higher dissociation rate of this simulation lowers the temperature and pressure difference across the transmitted shock, shifting the mechanical equilibrium of this feature further downstream. This figure highlights the coupling between the thermochemistry and fluid mechanics in this scenario.

**Fig. 7.**
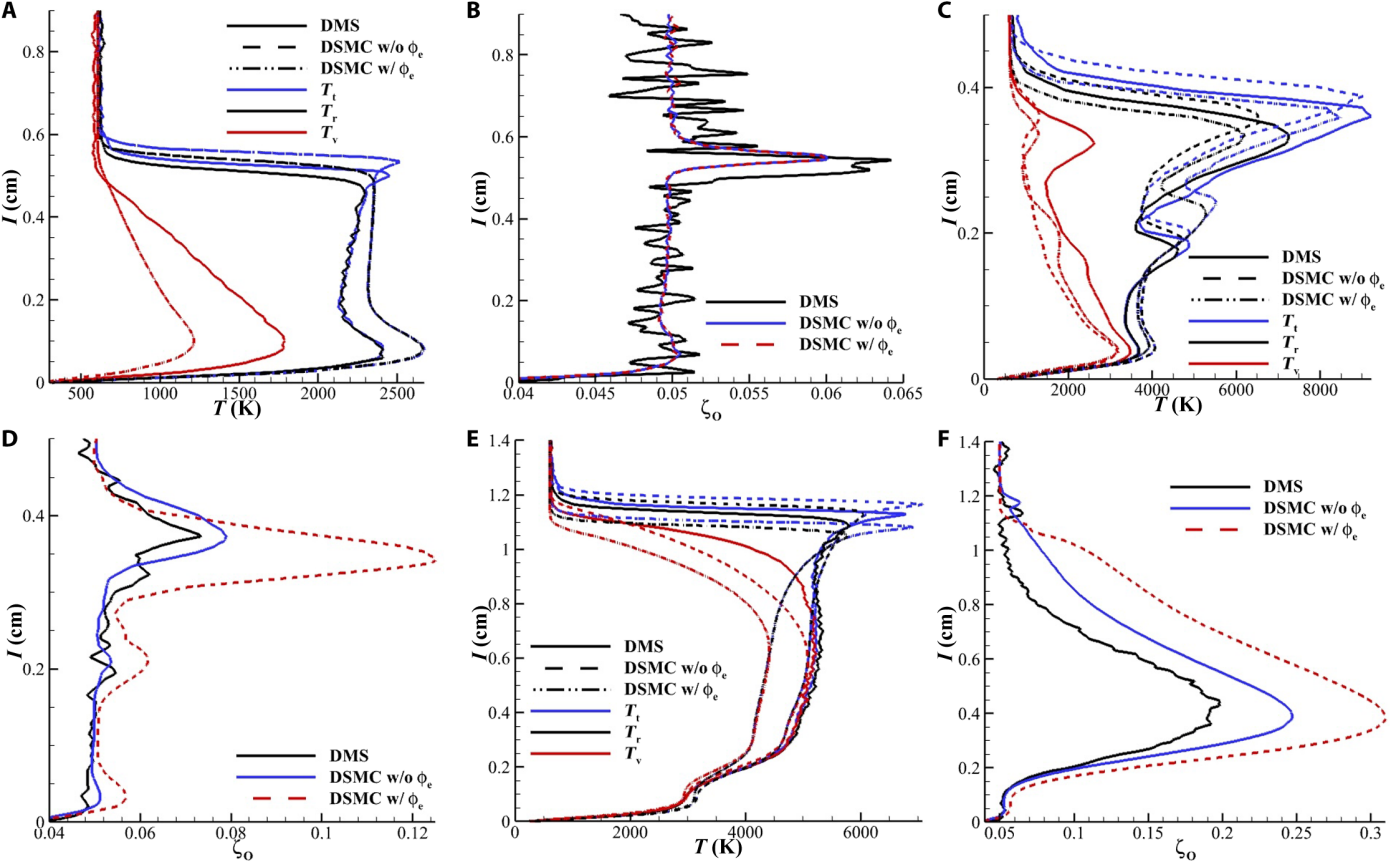
Comparison of temperature and composition profiles obtained from DMS and DSMC along select wall normal locations. This figure provided a more quantitative comparison of the thermodynamics state and chemical composition of the shock heated gas by analyzing the wall normal temperature and composition profiles and stations marked A, B, and C in Fig. 2A. (**A**) Temperature profiles at *X* = 6.88 cm along the article (line A in Fig. 2A). (**B**) Profiles of atomic oxygen mole fraction at *X* = 6.88 cm. (**C**) Temperature profiles along a wall normal passing through the triple point of the DMS (line B in Fig. 2A) and DSMC calculations. (**D**) Profiles of atomic oxygen mole fraction along a wall normal passing through the triple point of the simulations. (**E**) Temperature profiles at *X* = 12.17 cm along the article (line C in Fig. 2A). (**F**) Profiles of atomic oxygen mole fraction at *X* = 12.17 cm.

Figure 7 shows wall normal temperature and composition profiles along select stations marked A (*X* = 6.88 cm), B (triple point), and C (*X* = 12.17 cm) in Fig. 2A. Figure 7A show the wall normal temperature profiles at *X* = 6.88 cm. The translational temperature profile (*T*_t_) shows two peaks. The first peak at wall normal distance *l* = 0.55 cm corresponds to the location where the sample line intersects the oblique shock, and the second peak *l* = 0.1 cm shows the temperature associated with the gas heated from the stagnation region as it advects downstream. The DMS simulation predicts faster vibrational excitation than DSMC in this region. However, because of the lack of chemical reactivity in this region, both DSMC simulations provide identical results and the curves for temperature at this location, and these lines overlay each other. The comparatively faster relaxation of the vibrational mode of the DMS calculation leads to a smaller shock layer thickness for this simulation. Figure 7B shows the mole fraction of atomic oxygen at this location. This region of the flow has no dissociation occur. The variations in the atomic oxygen mole fraction occur at the free stream-shock interface and in the near wall region due to diffusion induced by sharp pressure and temperature gradients.

Figure 7C shows the temperature profiles for the three simulations along a wall normal passing through the triple point of each simulation. The first peak in the translational temperature profile corresponds to the temperature at the triple point, and the second peak corresponds to the location where the sample line intersects the reattachment shock. It is again observed that the vibrational relaxation in the DMS simulation is faster than the DSMC simulations. The DSMC simulation without ϕ_e_ predicts the same peak temperature as the DMS calculation. Figure 7D shows the atomic oxygen mole fraction at the triple point. While it is expected that the DSMC simulation with ϕ_e_ will have a higher mole fraction of atomic oxygen due to the elevated dissociation rate coefficient. The higher than expected atomic oxygen mole fraction for the simulation without ϕ_e_ can be attributed to the simplistic nature of the TCE model. More sophisticated models using DMS-tuned parameters will be able to provide a more favorable comparison. Figure 7E shows the temperature profiles along a wall normal at *X* = 12.17 cm on the second conic section (line C in Fig. 2A). Good agreement between temperature profiles of the DSMC simulation without ϕ_e_ and DMS is observed. Figure 7F shows the atomic mole fraction along this wall normal. A marginally higher atomic mole fraction is observed. As expected, the simulation with ϕ_e_ predicts higher dissociation and lower temperatures at the shock layer.

### Surface quantities

Pressure and heat load enacting on a hypersonic vehicle are key parameters used in vehicle design. In this section, we explore pressure loading and heat flux experienced by the test article’s surface. During the experimental run at the CUBRC facility (*20*), pressure transducers and thermocouples were used at discrete locations on the article. These data allow us to compare computational data with experimental observations. However, while making these comparisons, we have to acknowledge the experimental uncertainty associated with measurements, and tunnel test conditions factor into the experimentally reported data.

**Fig. 8.**
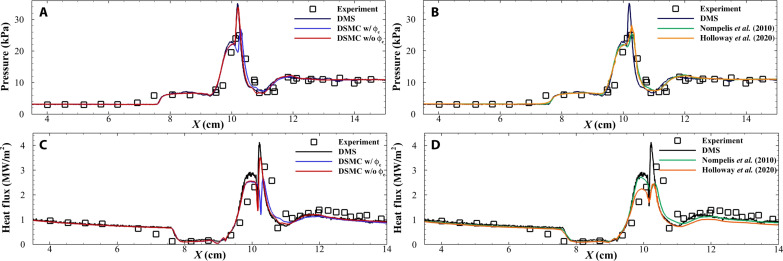
Comparison with experimental data. (**A**) Pressure at the article surface predicted by DMS, experimental data, and DSMC calculations presented in this article. (**B**) Compares pressure at the surface predicted by DMS with published Navier-Stokes CFD data of Nompelis *et al.* (*23*) and Holloway *et al.* (*25*). (**C**) Heat flux at the article surface predicted by DMS, experimental data, and DSMC calculations presented in this article. (**D**) Compares heat flux at the surface predicted by DMS with published Navier-Stokes CFD data of Nompelis *et al.* (*23*) and Holloway *et al.* (*25*).

Figure 8A compares the pressure load at the surface as predicted by DMS with experimental data. The DMS results agree well with the experimental data on the first conic section at *X* ≤ 7 cm. The pressure increase seen in the experimental data at ∼*X* = 7.5 cm and in the DMS data at ∼*X* = 7.7 cm corresponds to the separation foot. However, the DMS and experimental data agree with the pressure predicted in the separation region 7.7 cm ≤ *X* ≤ 9.5 cm. Downstream of this point the pressure increases as the flow is compressed by the reattachment shock, with the peak pressure load occurring at the point where the transmitted shock impinges on the article. The DMS result predicts the peak pressure at a location between two pressure transducers while agreeing well with the experimental profile. The pressure load drops as the flow expands downstream of the transmitted shock and levels off as the mechanical nonequilibrium in the flow dissipates. The DMS results agree well with the experimental data *X* > 11 cm.

Figure 8A also shows the comparison of surface pressure profiles obtained from the DSMC simulations discussed in the section above. Both DSMC solutions agree with the DMS calculation on the first conic section *X* < 9 cm and on the second conic section *X* > 11 cm. The key differences arise at the location and magnitude of the peak load. The DSMC simulation without ϕ_e_ provides an excellent comparison with the DMS result, with the peak pressure load value only 3% lower than the DMS solution. The solution with ϕ_e_ reports a peak pressure value 23% lower than the DMS value. Figure 8B provides a comparison of surface pressure profile with Navier-Stokes CFD data in (*23*) and (*25*). The DMS calculation agrees well with the CFD-predicted pressure on the first conic section, location of the separation and reattachment points of the flow, and pressure on the second conic section. The CFD data in (*23*) predict a peak pressure value 28% lower than the DMS result, while the data in (*25*) predict a 20% lower peak pressure.

Figure 8C shows heat flux computed with DMS and compares it with experimental data. The DMS calculation provides good comparison with experimental data on the first cone *X* ≤ 6.5 cm and the second cone after *X* ≥ 11 cm. The DMS solution predicts a peak heating of ∼4.1 MW/m^2^, the location of the DMS-calculated peak heat flux lies between the location of two sensors on the test article, and the experimentally observed maxima aligns with the spatial profile of the heat flux obtained using DMS. Figure 8C also shows the comparison of heat flux profiles obtained from the DSMC simulations discussed in the above. Both DSMC solutions agree with the DMS calculation on the first conic section *X* < 9 cm and on the second conic section *X* > 11 cm. The key differences arise at the location and magnitude of the peak load. The DSMC simulation without ϕ_e_ provides a reasonable comparison with the DMS result, with the peak heat flux value 3.5 MW/m^2^, which is ∼15% lower than the DMS solution. This discrepancy highlights that even with tuned rate coefficients and transport parameters, the simplicity of the TCE model can lead to a disagreement with DMS, emphasizing the need for more sophisticated models that can account for molecular level mechanisms discussed above. The solution with ϕ_e_ reports a peak heat flux of 2.6 MW/m^2^, which is ∼35% lower than the DMS value. The difference in peak heating between the two DSMC calculations is about ∼1 MW/m^2^. This difference emphasizes the effect of electronic excitation of oxygen on predictively determining heat flux.

Figure 8D provides a comparison of heat flux profile with published Navier-Stokes CFD data in (*23*) and (*25*). The CFD calculation in (*23*) predicts a higher heat flux under the reattachment shock instead of the point of impingement of the transmitted shock. The difference in heat flux at the point of transmitted shock impingement between DMS and (*23*) is 40%. CFD results in (*25*) predict the correct trend of the peak heat flux occurring at the point of impingement of the transmitted shock but underpredict the DMS data by 40%. Both CFD results underpredict heat flux when compared to the experimental data. Hence, the DMS and DSMC calculation with ϕ_e_ provide the best comparison with experimental data, pointing toward the lack of electronic excitation of oxygen in this case.

The trend of various flow solvers and models predicting similar surface quantities at *X* ≤ 7.5 cm and *X* ≥ 11 cm can be explained using Fig. 2A. Figure 2A shows the mach contour of the flow field along with select streamlines. It can be seen that gas interacting with the surface of the article originates on the first conic section, whereas any gas passing through the thermochemically active region of the detached shock is deflected around the article. The only features governed by thermochemistry that interact with the surface are the reattachment the transmitted shocks. Modulating the thermochemistry parameters and models alters the shock strength of the transmitted and reattachment shocks consequently, the peak heat flux and pressure loading are sensitive to numerical solvers and thermochemistry schemes. Therefore, with respect to the surface quantities, the key metric to assess the accuracy of a lower fidelity thermochemistry model with respect to this DMS calculation is the peak heat flux caused by the impingement of the transmitted shock onto the article surface.

## DISCUSSION

Here, we provide a first principles simulation of a near continuum hypersonic ground test. A Mach 8.2 oxygen flow over a double cone geometry, first studied experimentally at the CUBRC (*20*) facility as Run 88, is simulated using the ab initio DMS method. The DMS method embeds molecular interactions using ab initio interaction potentials within a time accurate flow field. Consequently, the only modeling input in DMS simulations is the quantum mechanically derived interaction potentials, and all flow field phenomena such as the transport, thermodynamic, and chemical properties of flow can be traced to the quantum mechanically derived PESs. In this work, we use a suite of 12 ab initio PESs (*30*–*32*) to fully simulate the dynamics of O_2_ + O_2_ and O_2_ + O interactions in the electronic ground state of collision partners. The conditions for this test generate a complex flow field with thermal and chemical nonequilibrium, making it ideal as a benchmark case to verify lower fidelity models.

The DMS calculation shows that in this scenario translational-rotational nonequilibrium only occurs at shock interfaces. Translational-vibrational nonequilibrium is observed in large portions of the flow, where the high velocity shock-heated gas experiences a slower local vibrational relaxation rate. Strong detached shocks and shock-shock interactions are observed to cause dissociation of molecular oxygen in the flow field. The fundamental nature of DMS enables molecular level analysis of the flow field. Particles flowing through select regions of the flow are studied to construct vibrational energy distributions. In vicinity of strong shocks and in regions of high dissociation, these energy distributions are observed to have non-Boltzmann characteristics, challenging simplistic formulations of thermochemistry models used in current flow solvers.

Two lower fidelity DSMC calculations are performed to study the impact of modulating the dissociation rate coefficient to account for electronic excitation of oxygen. The collision cross section and internal energy relaxation cross sections for these simulations were informed by prior DMS calculations (*19*, *56*, *57*, *59*). The total collision energy model is used such that the flow replicates ab initio rate coefficients produced by DMS. One simulation is run such that it does not account for electronically excited states of oxygen, while the rate coefficient on the second simulation is multiplied by a factor of ϕ_e_ = 16/3 to account for electronically excited states. As the DMS only uses PESs, corresponding to the electronic ground states of O_2_ and O, the DSMC simulation that does not account for electronically excited states is compared with DMS. It is observed that the tuned DSMC solutions provide good comparison with DMS. The tuned DSMC simulation predicts results within 3% for peak pressure and 14% for peak heat flux at the surface of the test article. Demonstrating how DMS can be used to verify lower fidelity solutions. The simulation that assumes full electronic excitation of molecular oxygen is used as a bounding case. This provides an insight into the effect of electronic excitation as the PESs needed to understand kinetics of electronic excitation of oxygen in shock-heated gases are not available in literature. It is observed that changing the dissociation rate coefficient to assume full electronic excitation moves the location of key flow features and results in peak pressure to be 23% and peak heat flux to be 35% lower than the DMS solution. The difference in peak heat flux between the two DSMC calculations is observed to be about 1 MW/m^2^. Such a large difference serves as a motivation for future work, highlighting the need to understand the mechanisms that cause electronic excitation in hypersonic flows.

Predicting the heat loads on a hypersonic vehicle’s surface is crucial to determining an optimal thermal protection strategy and therefore directly influences the safety and success of a mission. A comparison of heat flux at the test article’s surface between experimental data, DMS and DSMC calculations presented in this work, and published CFD data is presented. It is observed that published CFD work and the DSMC simulation that accounted for electronic excitation underpredict the heat flux at the surface, whereas DMS and DSMC simulation that did not account for electronic excitation agree best with experimental observations. Therefore, pointing toward a minimal excitation of the electronic internal energy mode in this scenario.

In general, in the past, DMS has been used to simulate unit problems to provide reaction rates (*52*–*57*) and define transport properties of gases (*19*, *59*), especially in temperature ranges where experimental data may be difficult to procure. However, this work demonstrates that with the increase in computational capacity, this methodology can be applied to large-scale problems to provide a fundamental understanding of molecular mechanisms prevalent in complex reacting flows. In the future, we hope that ab initio DMS calculations, such as the one presented here, can routinely use as anchor simulations across communities interested in modeling reactive and nonequilibrium flows.

## METHODS

### Direct simulation Monte Carlo

The DSMC method was originally proposed by Bird (*65*) as a stochastic approach to solve the Boltzmann equation. In a DSMC calculation, the simulation domain is discretized into cells the size of the local mean free path (λ_c_), and the simulation is progressed with time steps of the order of the smallest mean collision time in the flow field (τ_c_). Unlike deterministic molecular dynamics, only a representative subset of real particles in each control volume is simulated in the flow. The ratio of volumetrically accurate particles to simulated particles is the particle weight (*W*_p_). At every DSMC time step, the no time counter (*65*) algorithm is used to stochastically select particles in cell to undergo “collisions” to approximate the collision frequency of the gas. The particles selected for collision use a model to map the initial (reactant) to final (product) molecular states due to the collision. Over the decades, numerous empirical and semi-empirical collision models of varying fidelity have been proposed (*65*, *68*), particularly for internal energy relaxation (both rotational and vibrational modes) and chemical reactivity of the gas. Because of the computational cost associated with resolving length scales of the order of the mean free path, DSMC has largely only been applied to rarified problems. However, using high-performance computing resources DSMC has been successfully used to study continuum flows (*64*, *69*). For this article, the DSMC simulations have been carried out using the SPARTA code (*66*) developed at Sandia National Laboratories.

The SPARTA code has been used in two capacities in this article:

1) Foundation of DMS code: As stated above, DMS is the first principles variant of DSMC, where collision cross section models are replaced with molecular trajectory integration. We have implemented DMS routines in to the SPARTA code (*60*–*63*) to leverage the code’s efficient parallelization infrastructure. DMS method is discussed further in the subsection below.

2) DSMC calculations: Lower fidelity DSMC calculations are carried out as a point of comparison with the DMS solution. These calculations are used as an example on how tuning low fidelity models to first principles data can affect flow field properties. These calculations are performed using the variable hard sphere (VHS) viscosity cross section model with parameters proposed by Valentini *et al.* (*19*). To simulate the energy transfer between the translational and rotational modes, a constant rotational number of *Z*_r_ = 4 is used. This choice is justified as no rotational nonequilibrium is observed in the flow barring the shock interfaces. To simulate the energy transfer between the translational and vibrational modes, the native SPARTA model was tuned such that for O_2_ + O_2_ collisions, the simulation reproduces characteristic vibrational relaxation times proposed by Streicher *et al.* (*15*) and for O_2_ + O collisions the simulation reproduces characteristic vibrational relaxation times obtained from DMS (*57*). The parameters of the TCE model in SPARTA are modified to reproduce dissociation rate coefficients obtained from previous DMS studies (*57*). Details on the implementation of these models can be found in (*64*). All DMS-based coefficients used to tune the DSMC use the same PESs as the ones used in this study.

Reference (*57*) provides ab initio dissociation rate coefficients using the DMS method. Only PESs for the ground electronic states are currently available for O_2_ + O_2_ and O_2_ + O interactions (*30*–*32*). To compare with experimental shock tube data, (*57*) provides an argument that at extremely high temperatures (*T* > 5000 K), the shock-heated gas would have remained at a high temperatures for timescale sufficiently long to induce electronic excitation of molecular oxygen. Therefore, to compare dissociation rate coefficients with experimental data, the DMS rate coefficients are multiplied by a factor of ϕ_e_ = 16/3. In previous work, we analyzed CUBRC’s Run 88 only using dissociation rate coefficients that included the electronic excitation factor ϕ_e_ (*64*). In this work, we provide two DSMC solutions, one where the dissociation rates include the electronic excitation factor ϕ_e_ = 16/3 and one without.

The authors would like to point out that the TCE dissociation model used here is a fairly rudimentary dissociation model in DSMC. The DSMC results are included to show the effect of (i) tuning thermochemistry and transport coefficients and (ii) accounting for electronically excited states. There are many dissociation models available for DSMC in literature (*70*–*73*). As all models can be tuned to these coefficients, here, we only assess the effect of the coefficients. Evaluating the efficacy of various models is beyond the scope of this work.

### Direct molecular simulation

#### Overview

A detailed description of the DMS method can be found in (*48*), in this section, we provide a brief overview of the DMS method for clarity. As stated above, DMS is the first principles variant of DSMC. Similar to DSMC, the flow domain is discretized into cells, the size of the local mean free path (λ_c_), simulation is progressed with time steps of the order of the smallest mean collision time in the flow field (τ_c_), and a particle weight (*W*_p_) determines the ratio of actual particles to simulated particles in the simulation. The no time counter (NTC) algorithm (*65*, *68*) uses a collision cross section (σ) to determine collision pairs in each simulation cell. Unlike DSMC, (i) DMS particles have an internal structure, which is each atom of the simulated molecule is resolved and internal positions and velocity of bound atoms is stored. (ii) Instead of stochastic models determining the post-collision state of the colliding particles, in DMS, the particles selected for collision undergo a scattering trajectory calculation using appropriate PESs.

In DMS calculations, the hard sphere collision cross section used by the NTC algorithm is set such that $\sigma =\pi b_{\text{max}}^{2}$, where $b_{\text{max}}$ is the maximum impact parameter used to initialize trajectory calculations (*34*, *48*). Each collision pair is initialized for trajectory calculation by randomizing the molecule’s orientation, separating the particles by some conservative distance *D*_0_ and laterally displacing the particles by a distance *b*, which is randomized within the range of $0\leq b^{2}\leq b_{\text{max}}^{2}$. It has been shown that the exact value of the maximum impact parameter has no effect on a DMS solution, as long as the value is chosen conservatively (*48*). In this work, we use *b*_max_ = 10 Å. The particles are then assigned the relative velocity they had at the time of collision pair selection. The phase-space coordinates of all atoms comprising the colliding particles are marched forward using a velocity Verlet scheme (*74*), and the gradient of the PES provides the forces acting on the atoms. The time step for trajectory integration is set to Δ*t*_traj_ = 1 fs. The trajectory is integrated until the minimum separation between the atoms not bounded to the same molecule is greater than a cutoff distance *D*_0_, where forces experienced from the PES are negligible. In this work, we use *D*_0_ = 20 Å. The PES dictates whether a trajectory would yield an inelastic, elastic, or a reactive collision. After the trajectory is completed, each molecule is checked to establish if it is bound, quasi-bound, or dissociated. DMS results shown in this work are obtained by modifying the SPARTA DSMC code (*66*) to include internal structure for molecules and implementing trajectory integration routines as a separate collision model.

#### Potential energy surfaces

In this work, we explore a flow field that comprises of molecular and atomic oxygen. Molecular oxygen in its ground electronic state has two unpaired electrons, spin-coupled as a triplet. Therefore, O_2_ + O_2_ interactions can have an overall spin coupling of singlet, triplet, or quintet with statistical weights of these spin couplings being 1, 3, and 5, respectively. Three PESs, each corresponding to one of the spin couplings, are needed to fully simulate O_2_ + O_2_ in molecular oxygen’s ground electronic state. We use the set of three ab initio PESs developed in (*31*, *32*). When two oxygen atoms are selected for collision, one of the three O_2_ + O_2_ PESs is selected randomly, with the probability of the PES selection corresponding to their statistical weight: 1/9 for the singlet PES, 3/9 for the triplet PES, and 5/9 for the quintet PES. Similarly, atomic oxygen in its ground state also has two unpaired electrons, and O_2_ + O interactions can also occur with the total spin coupling being singlet, triplet, or quintet. In addition, the spatial symmetry of atomic oxygen generates a threefold degenerate ground state for all three spin states. Hence, because the spin and spatial degeneracies, nine unique PESs are required to fully describe O_2_ + O interaction in the ground states of the collision partners. In this work, we use a suite of nine adiabatic ab initio PESs developed in (*30*). When an O_2_ + O collision occurs in the DMS flow, one of the nine PESs is selected randomly with the probability of the PES selection corresponding to their statistical weight: 1/27 for each singlet PES, 3/27 for each triplet PES, and 5/27 for each quintet PES. O + O interactions are simulated using the diatomic energy curve of molecular oxygen in (*30*). Table S2 shows the diatomic characteristics of molecular oxygen obtained from the PESs.

**Fig. 9.**
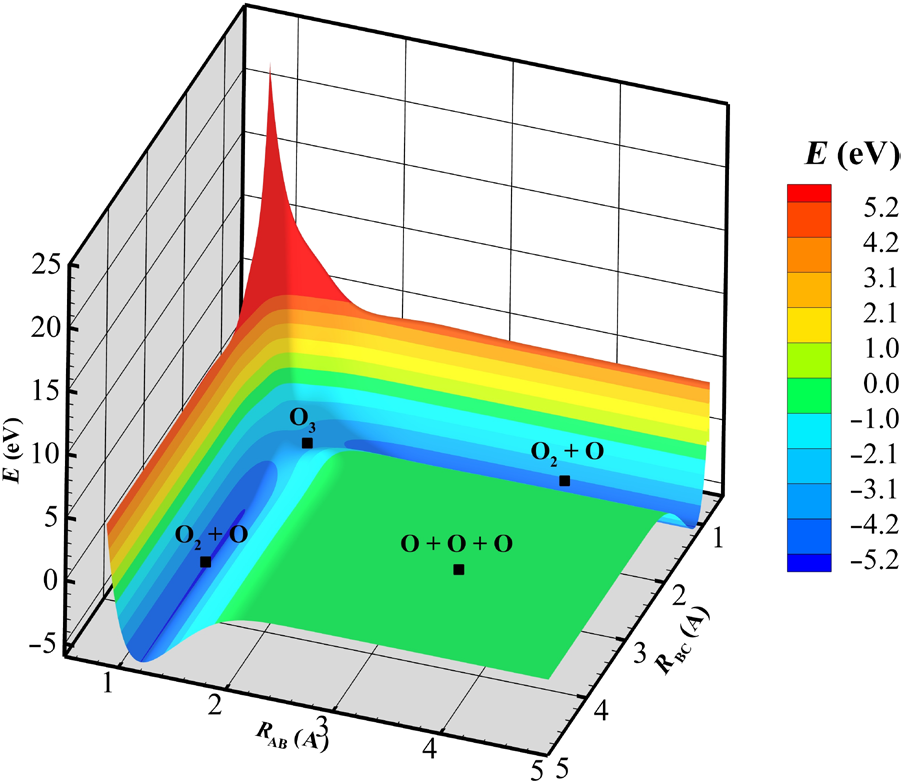
Visualization of the PES for three oxygen atoms (O*_A_*, O*_B_*, O*_C_*) in a linear configuration. The *x* axis represents distance between O*_A_* and O*_B_* in angstroms, *y* axis represents distance between O*_B_* and O*_C_* in angstroms, and the *z* axis represents the potential energy of the system.

Figure 9 shows a visualization of one of the nine PESs (1^1^A′ state) (*30*, *56*) used to define O_2_ + O collisions. In this visualization, the three oxygen atoms (O*_A_*, O*_B_*, and O*_C_*) are in a linear configuration. The *x* axis represents distance between O*_A_* and O*_B_*, *y* axis represents distance between O*_B_* and O*_C_*, the *z* axis represents the potential energy of the system. The gradient of the potential energy dictates the forces acting on the atoms and hence the trajectory of an O_2_ + O collision. Four key features of the PES topography help visualize the collision configurations and outcomes. (i) When any one of the two interatomic distances gets small (approximately ≤1 Å), there is a marked increase in potential energy of the system—these features are referred as the repulsive wall. (ii) When one of the interatomic distances is close to the equilibrium bond length of diatomic oxygen (∼1.2 Å) and the second distance is larger we get a stable O_2_ + O configuration, with the well-depth potential energy corresponding to the O_2_ bond energy of 5.21 eV. (iii) When both distances are ∼1.5 Å, we see a metastable O_3_ configuration with a potential energy of ∼−3 eV. (iv) Last, when all interatomic distances are large, we get a noninteracting O + O + O configuration, with a potential energy of zero.

#### Treatment of internal energy

DMS calculations do not assume any decoupling between rovibrational modes of a molecule as the trajectory calculation routines only use the internal position and velocity of bound atoms. To provide an analog to traditional models in DSMC and CFD that use “rotational” and “vibrational” energy modes, the internal rovibrational energy of a molecule is calculated from the internal position and velocity of bound atoms and split into rotational energy and vibrational energy as a post-processing step. In this work, we use the vibrational prioritization framework (*29*, *75*) to divide internal rovibrational energy [ε(v,j)] into rotational energy [$\varepsilon_{r}\left(j\right)$] and vibrational energy [$\varepsilon_{v}\left(v\right)$]. Under this methodology, first, the internal energy [ε(v,j)] is mapped to a rovibrational state with a vibrational level “v” and rotational level “j.” The molecule is first assigned the vibrational energy of level “v” corresponding to rotational level j=0

$$\varepsilon_{v}\left(v\right)=\varepsilon_{\text{int}}\left(v,0\right)$$ (2)

The remaining internal energy is assigned to the rotational mode

$$\varepsilon_{r}\left(j\right)=\varepsilon_{\text{int}}\left(v,j\right)-\varepsilon_{\text{int}}\left(v,0\right)$$ (3)

The average rotational and vibrational energies of molecules in a simulation cell are used to define rotational and vibrational temperatures of the cell. The rotational temperature is calculated as

$$T_{r}=\frac{\langle \varepsilon_{r}\rangle }{k_{B}}$$ (4)

Because of assumptions made in Eq. 2 and the anharmonicity of the diatomic potential, at higher temperatures, $\langle \varepsilon_{v}\rangle /k_{B}>T_{v}$. Figure 9 shows the average vibrational energy obtained from a continuous distribution using the above splitting mechanism at a given temperature. To account for this effect, we propose a simple polynomial fit to the curve shown in Fig. 9 (listed in the Supplementary Materials), mapping the average vibrational energy of the system to a vibrational temperature in Eq. 5. This fitting function is trained on a dataset of $T_{v}\in$ (300 K, 12,000 K) with a root mean squared error of 0.58 K. For the scenario discussed in this paper, the vibrational temperature varies in the range $T_{v}\in$ (300 K, 6000 K)

$$\begin{array}{c} T_{v}=1.2495\times 10^{-25}{\left(\frac{\langle \varepsilon_{v}\rangle }{k_{B}}\right)}^{7}-6.4568\times 10^{-21}{\left(\frac{\langle \varepsilon_{v}\rangle }{k_{B}}\right)}^{6}\\ +1.3175\times 10^{-16}{\left(\frac{\langle \varepsilon_{v}\rangle }{k_{B}}\right)}^{5}-1.2256\times 10^{-12}{\left(\frac{\langle \varepsilon_{v}\rangle }{k_{B}}\right)}^{4}\\ +5.45822\times 10^{-9}{\left(\frac{\langle \varepsilon_{\text{vib}}\rangle }{k_{B}}\right)}^{3}-2.4941\times 10^{-5}{\left(\frac{\langle \varepsilon_{v}\rangle }{k_{B}}\right)}^{2}\\ +1.0127\left(\frac{\langle \varepsilon_{v}\rangle }{k_{B}}\right)+49.169 \end{array}$$ (5)

Note that the vibrational prioritization framework is one of many ways to divide rovibrational energy of a molecule into rotational and vibrational modes but is commonly used in computational chemistry. Procedures discussed in this subsection are strictly a post-processing steps aimed to provide analogous terms to commonly used definitions in the nonequilibrium CFD community. The authors would like to stress that DMS calculations operate solely on atomic positions and velocities. The definitions of $\varepsilon_{v}$, $\varepsilon_{r}$, $T_{v}$, and $T_{r}$ do not feed into the calculations in any form and hence have no impact on the DMS solution.
