# Supplementary material for: Ab initio investigation of a hypersonic double cone experiment
Source: Sci Adv. 2025 Feb 5;11(6):eads2147. doi: 10.1126/sciadv.ads2147 (PMC11797544; doi:10.1126/sciadv.ads2147)
Supplement: Supplementary file 1 — Fig. S1 Tables S1 and S2 [file sciadv.ads2147_sm.pdf]

Science Advances
AAAS

Supplementary Materials for

Ab initio investigation of a hypersonic double cone experiment

Maninder S. Grover *et al.*

Corresponding author: Maninder S. Grover, mgrover1@udayton.edu

Sci. Adv. **11**, eads2147 (2025)
DOI: 10.1126/sciadv.ads2147

This PDF file includes:

Fig. S1
Tables S1 and S2

Supplementary Materials For

Ab Initio Investigation of a Hypersonic Double Cone Experiment

Grover *et al.* *

∗Corresponding author. Email:mgrover1@udayton.edu

Figure S1 shows the variation of mean vibrational energy as a function of temperature for diatomic oxygen.

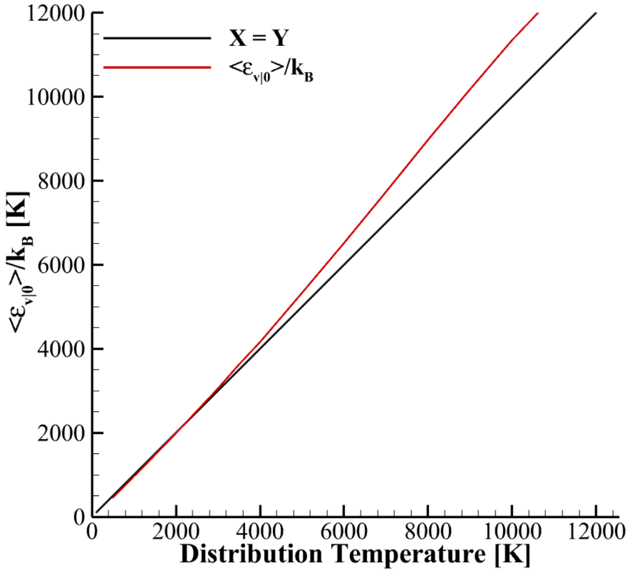

Figure S1: Variation of average vibrational energy as a function of temperature for molecular oxygen.

Table S1 lists properties of diatomic oxygen obtained from the *ab initio* PES.

Dissociation energy at j=0	5.21 eV
Number of vibrational levels for j=0	45
Number of rotational levels for v=0	241
Number of ro-vibrational levels	6115
Number of bound ro-vibrational levels	4581
Number of quasi-bound ro-vibrational levels	1534

Table S1: Properties of diatomic oxygen from the *ab initio* potential energy surface.

Table S2 lists the vibrational ladder at J = 0. This vibrational binning is used to obtain vibrational distribution functions discussed in the article.

Vibrational Level	Vibrational Energy [eV]
0	0.098213
1	0.29222
2	0.48303
3	0.67068
4	0.85522
5	1.0367
6	1.2152
7	1.3907
8	1.5633
9	1.733
10	1.8999
11	2.064
12	2.2252
13	2.3837
14	2.5394
15	2.6923
16	2.8424
17	2.9897
18	3.1341
19	3.2755
20	3.414
21	3.5494
22	3.6816
23	3.8105
24	3.9361

Vibrational Level	Vibrational Energy [eV]
25	4.0581
26	4.1763
27	4.2907
28	4.4009
29	4.5067
30	4.6078
31	4.7038
32	4.7942
33	4.8785
34	4.9559
35	5.0254
36	5.0856
37	5.1345
38	5.1685
39	5.1861
40	5.1968
41	5.2043
42	5.2088
43	5.2108
44	5.2112

Table S2: Properties of diatomic oxygen from the potential energy surfaces.
